# Identification of the role of a MAP kinase Tmk2 in *Hypocrea jecorina* (*Trichoderma reesei*)

**DOI:** 10.1038/srep06732

**Published:** 2014-10-23

**Authors:** Mingyu Wang, Yanmei Dong, Qiushuang Zhao, Fangzhong Wang, Kuimei Liu, Baojie Jiang, Xu Fang

**Affiliations:** 1 State Key Laboratory of Microbial Technology, School of Life Sciences, Shandong University, Jinan 250100, China; 2 National Glycoengineering Research Center, Shandong University, Jinan 250100, China

## Abstract

Despite the important role of MAPKs in signal transduction, their functions in the cellulase hyper-producing filamentous fungus *Hypocrea jecorina* haven't been studied except for the Hog1-like Tmk3. In this work, we constructed and explored the features of *H. jecorina Δtmk2* to identify the role of this Slt2-homologous Tmk2. It is suggested from the results that Tmk2 is involved in cell wall integrity, sporulation and cellulase production. Although bearing similar roles in cell wall integrity maintenance, Tmk2 and Tmk3 appear to also have distinct functions: Tmk3 participates in high osmolarity resistance while Tmk2 does not; Tmk2 participates in sporulation but not Tmk3; Tmk3 is involved in promoting cellulase production while Tmk2 is involved in repressing cellulase formation. These studies provide the first insight into the function of Tmk2 in *H. jecorina* and contribute to understanding the signal transduction processes leading to the regulation of cellulase production in this important cellulase hyper-producer.

H*ypocrea jecorina* (syn. *Trichoderma reesei*) is one of the most important cellulase-producing industrial filamentous fungal species^1^. Isolated during World War II, it has received many rounds of improvement over the past 70 years to gain the cellulase hyper-secreting capability: modern industrial *H. jecorina* strains can usually produce over 20 g/L proteins^2^. This unusual feature has made *H. jecorina* one of the model cellulase-producing organisms for the understanding of cellulase production regulation and other prominent physiological processes in filamentous fungi.

The mitogen-activated protein kinase (MAP kinase, MAPK) pathways are among the most well-known signal transduction pathways in eukaryotes^3^. These pathways feature signal relay cascades involving three kinases: MAP kinase kinase kinase (MAPKKK), MAP kinase kinase (MAPKK) and MAP kinase^4^. These three kinases collaborate to pass phosphorylation states (the signal) from upstream sensors to MAPKKK, to MAPKK, to MAPK, and eventually to downstream elements such as transcription factors for the regulation of physiological functions. In filamentous fungi, three MAPKs have been identified, respectively homologous to yeast Hog1, Slt2 and Fus3^5^,^6^.

Investigations on Hog1 homologues in *Aspergillus nidulans*^7^, *Magnaporthe grisea*^8^, *Cryphonectria parasitica*^9^, *Neurospora crassa*^10^, *Trichoderma harzianum*^11^, and *Aspergillus fumigatus*^12^ showed their involvement in adaptation to high osmotic pressure, a conserved role of Hog1 in both filamentous fungi and yeasts^13^,^14^. Hog1 homologues were also shown to be involved in other species-specific processes such as sporulation^8^,^9^, oxidative stress response^12^, thermal stress response^15^, tolerance to heavy metals^11^ and pigmentation^9^.

Investigations in filamentous fungi including *Beauveria bassiana*^16^, *Fusarium graminearum*^17^, *N. crassa*^18^, *A. fumigatus*^19^, *Magnaporthe oryzae*^20^, *Claviceps purpurea*^21^, *Trichoderma virens*^22^, *Mycosphaerella graminicola*^23^, *A. nidulans*^24^, and *Coniothyrium minitans*^25^ suggested Slt2 homologues primarily participate in cell wall integrity maintenance similarly to the budding yeast *Saccharomyces cerevisiae*^26^. Exceptions, however, do exist. For instance, deletion of *slt2*-like genes in *Botrytis cinerea*^27^ and *Colletotrichum lagenarium*^28^ didn't lead to weakened cell wall structure. Other pathways in which Slt2-homologues function include virulence^16^,^17^,^20^,^29^, sporulation^17^,^18^,^25^,^27^,^29^, female fertility^17^,^18^, secondary metabolism^18^,^29^,^30^, hyphal polarity maintenance^18^,^19^,^31^, surface hydrophobicity maintenance^16^,^22^,^29^, stress response^19^ and circadian rhythm maintenance^32^.

The functions of Fus3 homologues in filamentous fungi are quite diverse, out of which the participation in the secretion of extracellular proteins is of particular interest, exemplified in *T. virens*^33^,^34^ and *Trichoderma atroviride*^35^.

Despite the importance of *H. jecorina* in the understanding of fungal physiology, experimental investigations on its signal transduction pathways are quite limited^5^. In particular, signal transduction pathways transmitting external signals for the regulation of cellulase production, the most important physiological process in this cellulase hyper-producing species, are yet to be well understood. Previous *in silico* reconstruction of signal transduction pathways of *H. jecorina* suggested the presence of three MAPKs: Tmk1 that is homologues to yeast Fus3, Tmk2 that is homologues to yeast Slt2 and Tmk3 that is homologous to yeast Hog1^1^. The functions of these MAPKs in *H. jecorina* were unknown before research carried out in our laboratory showed Tmk3 functions in cell wall integrity maintenance, high osmolarity resistance, biosynthesis and cellulase production regulation^5^. The unusual role of Tmk3 in cell wall integrity maintenance and the interesting participation of Tmk3 in cellulase production regulation lead to three questions: 1) since the Hog1-like Tmk3 acts similarity to Slt2-type MAPK in cell wall integrity maintenance, what is the role of the Slt2-like Tmk2? 2) Is Tmk2 simply a redundancy to Tmk3? 3) Does Tmk2 also regulate cellulase production and how? In this study, with these questions in mind, we attempted to identify the role of Tmk2 in *H. jecorina*, and to compare the functions of Tmk2 and Tmk3 in this very important cellulase-producing model organism.

## Results

### Construction, growth and sporulation of *H. jecorina Δtmk2*

**Figure 1:**
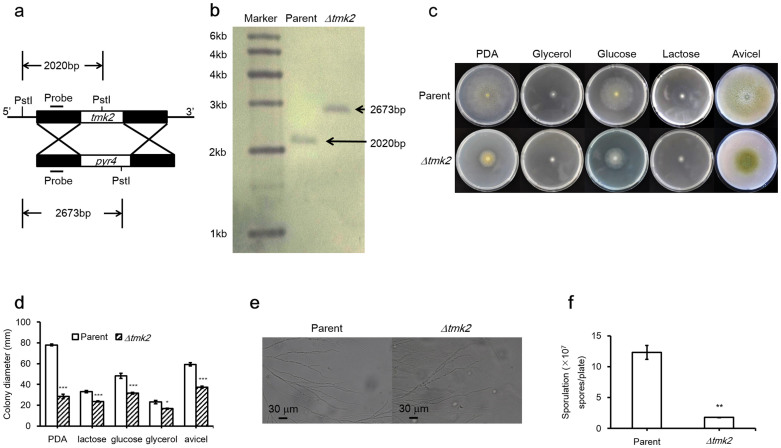
Growth of *H. jecorina* parent and *Δtmk2* strains on plates, sporulation and microscopic observations of hyphae. Panel a, schematic representation of *tmk2* deletion. Panel b, Southern blotting analysis of *H. jecorina* parent and *Δtmk2* strains. Panel c, *H. jecorina* parent and *Δtmk2* strains grown on PDA plates, Avicel double layer plates and minimal media plates containing various carbon sources. Gly, glycerol; Glu, glucose; Lac, lactose; Avi, Avicel. Panel d, colony diameters of *H. jecorina* parent and *Δtmk2* strains grown on PDA plates, Avicel double layer plates and minimal media plates containing various carbon sources. Panel e, microscopic images of hyphae from *H. jecorina* parent and *Δtmk2* strains. Panel f, sporulation of *H. jecorina* parent and *Δtmk2* strains grown on PDA plates. * P<0.05; ** P<0.01; *** P<0.001.

Sequence comparison between Slt2 from *S. cerevisiae* and Tmk2 from *H. jecorina* showed 69% sequence identity, supporting the previous suggestion that Tmk2 encodes a Slt2-type MAPK^5^ (see Supplementary Information 1 online for sequence alignment). *H. jecorina Δtmk2* was constructed by homologous recombination using *H. jecorina* *Δku70* as the parent strain, whose non-homologous end joining pathway was disrupted (Fig 1a–b). Both *H. jecorina* parent and *Δtmk2* strains were grown on PDA plates, minimal media plates containing glucose, glycerol, or lactose as carbon sources, as well as Avicel double layer plates. A reduction of growth on agar plates was observed for *H. jecorina Δtmk2* in comparison to the parent strain (Fig. 1c–d). To examine whether deletion of *tmk2* leads to phenotypic changes of hyphae, spores of both *H. jecorina* parent and *Δtmk2* strains were inoculated on glass slides on which a thin layer of PDA agar media was overlaid. The slides were incubated for two days to allow hyphal growth and were subsequently observed with a microscope. As is shown in Figure 1e, no apparent changes of hyphae morphology were observed, suggesting deletion of *tmk2* doesn't substantially impact hyphae development in *H. jecorina*.

Sporulation levels of *H. jecorina* parent and *Δtmk2* strains were assayed by inoculating the same amount of spores (1 × 10^5^) on PDA plates and comparing the level of spore production after 6 days of growth. A significant smaller amount of spores was produced by *H. jecorina Δtmk2* (1.78 ± 0.04 × 10^7^/plate, mean ± s.d., n = 9) than the parent strain (1.23 ± 0.11 × 10^8^/plate, mean ± s.d., n = 9, P = 0.0038, Fig. 1f). These results lead to the suggestion that deletion of *tmk2* results in reduced sporulation in *H. jecorina*.

### Involvement of Tmk2 in cell wall integrity maintenance

**Figure 2:**
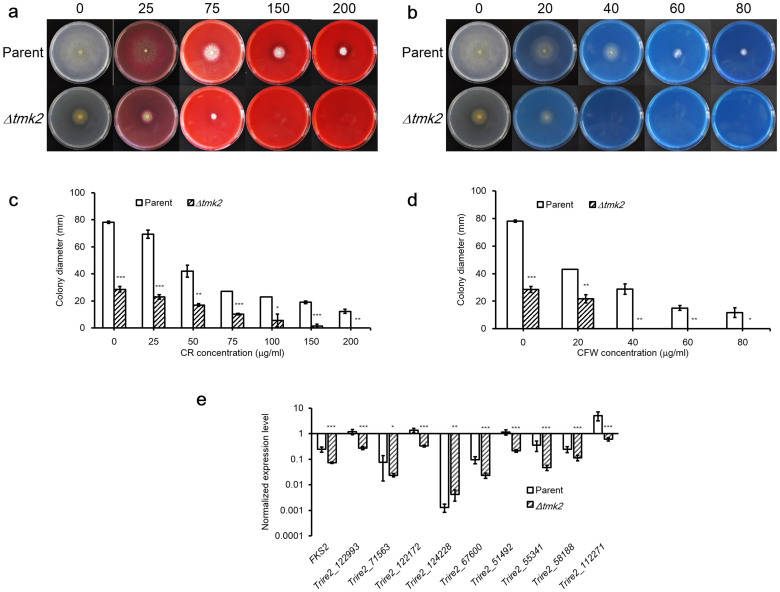
Involvement of *H. jecorina* parent and *Δtmk2* strains in cell wall integrity maintenance. Panel a, growth of *H. jecorina* parent and *Δtmk2* strains on PDA plates containing various concentrations of CR. Numbers above the plates indicate concentrations of CR in each plate (in μg/ml). Panel b, growth of *H. jecorina* parent and *Δtmk2* strains on PDA plates containing various concentrations of CFW. Numbers above the plates indicate concentrations of CFW in each plate (in μg/ml). Panel c, colony diameters of *H. jecorina* parent and *Δtmk2* strains grown on PDA plates containing various concentrations of CR. Panel d, colony diameters of *H. jecorina* parent and *Δtmk2* strains grown on PDA plates containing various concentrations of CFW. Panel e, normalized expression levels of chitin synthase- and β-1,3-glucan synthase-coding genes. The expression levels of each gene were compared to the expression levels of Actin-coding gene for normalization. * P<0.05; ** P<0.01; *** P<0.001.

The sensitivity of *H. jecorina* parent and *Δtmk2* strains to cell wall interfering substances Congo Red (CR) and Calcofluor White (CFW) was tested, in order to find out if deletion of *tmk2* leads to defective cell wall. The parent strain could tolerate at least 200 μg/ml CR or 80 μg/ml CFW, while the *Δtmk2* strain was unable to grow on plates containing 150 μg/ml CR or 40 μg/ml CFW (Fig. 2a–2d). These results lead to the suggestion that deletion of *tmk2* results in increased sensitivity to cell wall interfering substances.

We further analyzed the transcription of chitin synthase coding genes in *H. jecorina* parent and *Δtmk2* strains to see if Tmk2 control the synthesis of chitin, a key component of cell wall. From the sequenced genome of *H. jecorina*, we were able to identify 9 chitin synthase coding genes (Trire2_112271, Trire2_58188, Trire2_55341, Trire2_51492, Trire2_124228, Trire2_122172, Trire2_71563, Trire2_67600, Trire2_122993) and one β-1,3-glucan synthase coding gene (Trire2_78176, *fks*), respectively involved in the synthesis of chitin and β-1,3-glucan, two major components of fungal cell wall. Real-time PCR analysis of the transcriptional levels of these genes in both *H. jecorina* parent and *Δtmk2* strains showed they are clearly and significantly downregulated in *H. jecorina Δtmk2* (Fig. 2e) except for Trire2_124228. Respectively, Trire2_112271, Trire2_58188, Trire2_55341, Trire2_51492, Trire2_122172, Trire2_71563, Trire2_67600, Trire2_122993 and *fks* were downregulated by 8.6 (P = 1.1 × 10^−4^, n = 9), 2.1 (P = 2.2 × 10^−4^, n = 9), 7.6 (P = 3.0 × 10^−4^, n = 9), 5.4 (P = 5.2 × 10^−6^, n = 9), 4.2 (P = 1.8 × 10^−6^, n = 9), 3.2 (P = 0.035, n = 9), 4.2 (P = 8.6 × 10^−5^, n = 9), 4.3 (P = 6.6 × 10^−6^, n = 9) and 3.3 (P = 9.8 × 10^−6^, n = 9) folds.

### Involvement of Tmk2 in cellulase production

**Figure 3:**
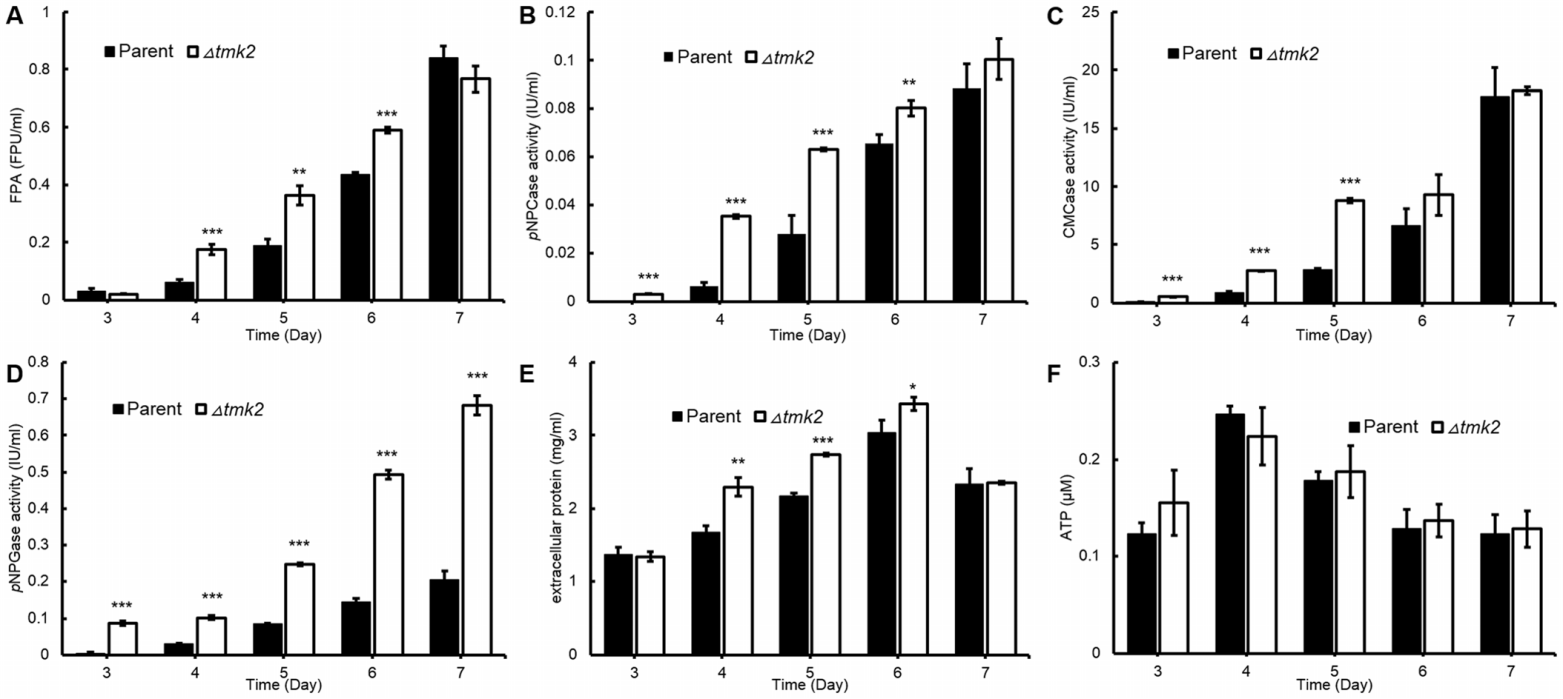
Cellulase production and biomass accumulation of *H. jecorina* parent and *Δtmk2* strains. Panel a, extracellular protein secretion levels. Panel b, FPA levels. Panel c, abilities to hydrolyze *p*NPC (*p*NPCase activity levels). Panel d, abilities to hydrolyze CMC (CMCase activity levels). Panel e, abilities to hydrolyze *p*NPG (*p*NPGase activity levels). Panel f, biomass measured by ATP concentration in cultures. * P<0.05; ** P<0.01; *** P<0.001.

We assayed extracellular cellulase production in both *H. jecorina* parent and *Δtmk2* strains. The cellobiohydrolase, endoglucanase and β-glucosidase activities were respectively measured by abilities to hydrolyze *p*-nitrophenyl-β-_D_-cellobioside (*p*NPC), carboxymethylcellulose (CMC) and *p*-nitrophenyl-β-_D_-glucopyranoside (*p*NPG). The overall cellulase activity was measured by the Filter Paperase Activity (FPA). As is shown in Figure 3a–e, higher cellulase production levels could be identified in the *Δtmk2* strain: out of all 25 data sets (5 days ×5 activities/concentrations), 18 are statistically significantly different (n = 3, P<0.05) between *H. jecorina* parent and *Δtmk2* strain. We further measured the ATP level in cultures according to previous suggestions and used it to represent the cellular biomass in the cultures^36^. As is shown in Figure 3f, the ATP accumulation in *H. jecorina* parent and *Δtmk2* is not statistically different in submerged cultures used for cellulase production analysis, therefore leading to the suggestion that *H. jecorina Δtmk2* produces more cellulase per unit of biomass than the parent strain.

**Figure 4:**
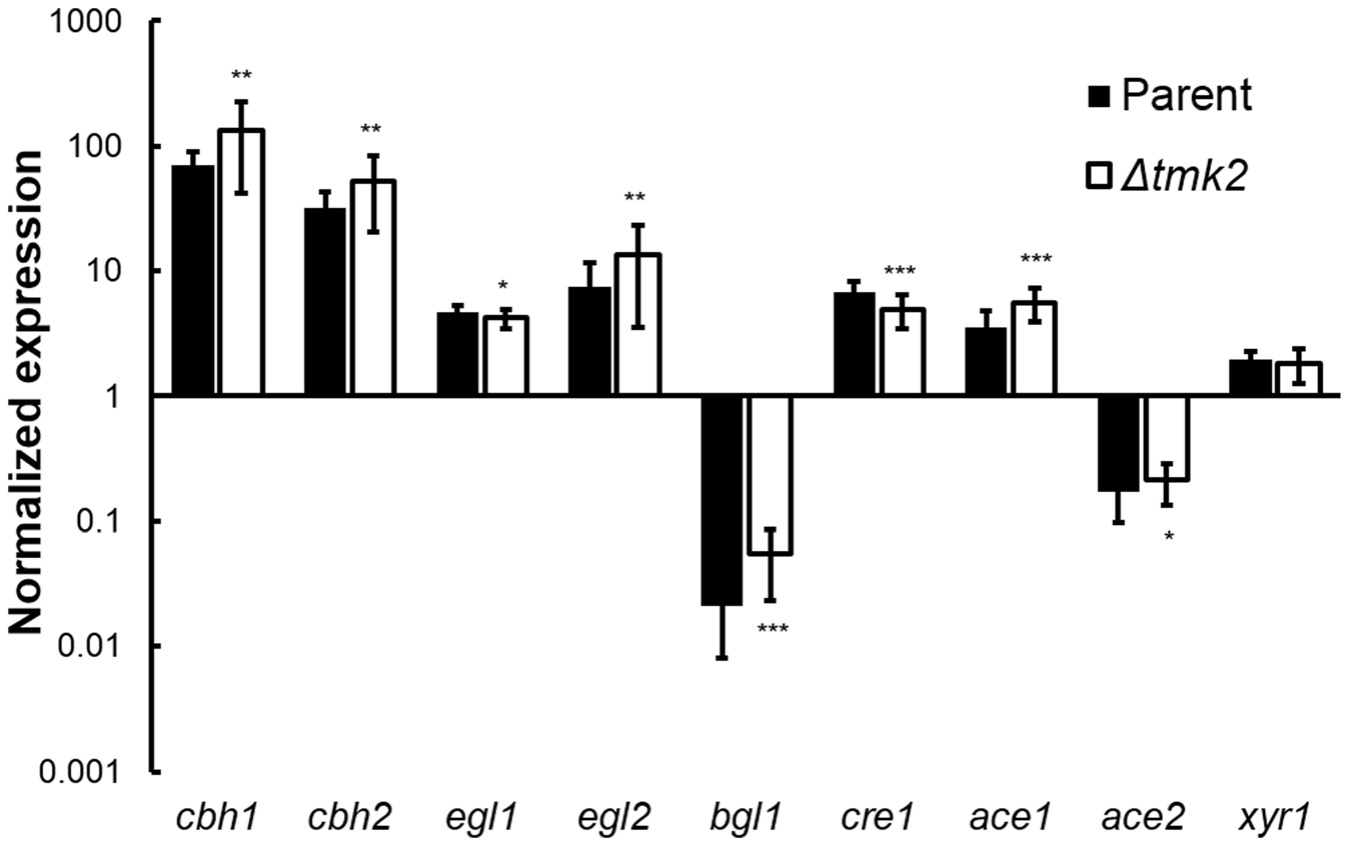
Transcriptional abundance of cellulase- and transcription factor-coding genes. The expression levels of each gene were compared to the expression levels of Actin-coding gene for normalization. * P<0.05; ** P<0.01; *** P<0.001.

The transcriptional levels of *cbh1*, *cbh2*, *egl1*, *egl2*, *bgl1*, *cre1*, *ace1*, *ace2* and *xyr1*, respectively coding cellobiohydrolase I, cellobiohydrolase II, endoglucanase I, endoglucanase II, β-glucosidase I, and known transcription factors that regulate cellulase expression were analyzed using qPCR in both *H. jecorina* parent and *Δtmk2* strains. Although statistical tests suggested the upregulation or downregulation of all these genes but *xyr1* in *H. jecorina Δtmk2* in comparison with the parent strain, the difference between the two strains is too minor to conclude a biologically significant difference (Fig. 4). A suggestion can therefore be made that the modulation of Tmk2 on cellulase production is not on the transcriptional level.

### Tmk2 is not involved in high osmolarity resistance or oxidative stress response

**Figure 5:**
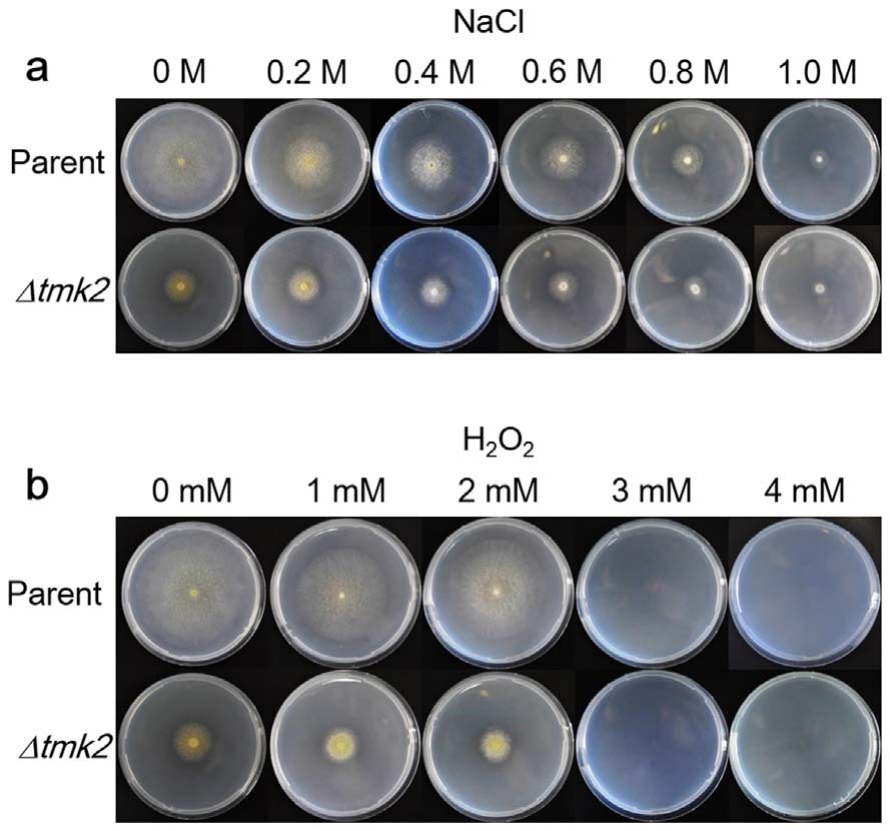
Growth of *H. jecorina* parent and *Δtmk2* strains on plates containing NaCl and H_2_O_2_. Panel a, growth on plates containing NaCl. Panel b, growth on plates containing H_2_O_2_.

Both *H. jecorina* parent and *Δtmk2* strains were grown on PDA plates containing NaCl or H_2_O_2_ (Fig. 5). Both strains could grow on all the plates containing NaCl. The ratios of colony diameters between *H. jecorina Δtmk2* and parent strain didn't decrease when NaCl concentrations increased: the ratios were respectively 0.37, 0.51, 0.62, 0.71, 0.79 and 0.81 for plates containing 0 M, 0.2 M, 0.4 M, 0.6 M, 0.8 M and 1 M NaCl. These results suggested there is no apparent increase of sensitivity to higher contents of NaCl following *tmk2* deletion, and further suggested Tmk2 is not involved in combating high osmolarity. Both *H. jecorina* parent and *Δtmk2* strains could grow on plates containing 1 mM or 2 mM H_2_O_2_, but neither strains could grow on plates containing 3 mM or 4 mM H_2_O_2_ (Fig. 5). Similarly to NaCl-containing plates, the ratios of colony diameters between *H. jecorina Δtmk2* and parent strains didn't decrease when H_2_O_2_ concentrations increased: 0.37 for plates containing 0 mM H_2_O_2_, 0.45 for plates containing 1 mM H_2_O_2_, and 0.48 for plates containing 2 mM H_2_O_2_, suggesting the sensitivity to H_2_O_2_ didn't increase after *tmk2* deletion and that Tmk2 is not involved in combating oxidative stress.

## Discussion

The improved sensitivity of *H. jecorina Δtmk2* to cell wall interfering substances, a typical phenomenon in cell wall defective strains, as well as the hampered transcription of chitin synthases in *H. jecorina Δtmk2* lead to the suggestion that Tmk2 is involved in cell wall integrity maintenance in *H. jecorina*. This physiological role is not a surprise, because Slt2 homologues have been found to participate in this process in many other filamentous fungi such as *T. virens*^22^ and *A. fumigatus*^19^. The observation in this (Fig. 2) and previous work^5^ that both Tmk2 (Slt2-type MAPK) and Tmk3 (Hog1-type MAPK) are involved in cell wall integrity pathway, however, was novel in filamentous fungi. Our results cannot lead to the suggestion of the relationship between the two MAPKs in cell wall integrity pathway, but it is not uncommon that two different MAPKs play similar roles in the same organism. For instance, both Fus3 and Slt2 homologues in *Cochliobolus heterostrophus* are implicated to participate in conidiation, surface hydrophobicity maintenance and virulence^29^,^37^. Possible explanations to this phenomenon include the redundancy of signal transduction pathways to key physiological functions or the ability to receive the same upstream phosphorylation signals for structurally similar proteins (Tmk2 and Tmk3 share 43% sequence identity, see Supplementary Information 2 online for sequence alignment). It cannot be ruled out, however, that these MAPKs sharing similar roles play different important roles in the same pathway. All these possibilities need to be examined in more future in-depth investigations to further clarify the relationship between different MAPKs.

Cellulase production is a complicated process that is influenced by many external factors. Carbohydrate signals have a profound impact on cellulase production: quite a few di- and poly-saccharides such as cellulose, lactose and sophorose induce cellulase production^38^,^39^, while glucose represses cellulase production^38^. Light was shown to stimulate cellulase production in the model organism *H. jecorina*^40^. Investigations on signal transduction pathways that transmit these external signals to nucleus have helped to identify several key factors. For instance, G proteins, a PAS/LOV domain protein ENVOY and protein kinase A have been shown to be involved in light-regulated cellulase production^41^,^42^,^43^,^44^. It is a reasonable proposal that these external signals eventually determine phosphorylation states of transcription factors that regulate cellulase transcription and eventually lead to the modulation of cellulase production. Quite a few transcription factors have been shown to regulate the transcription of cellulase-coding genes. The most well studied transcription factors include activators Xyr1^45^, XlnR^46^, ACEII^47^, Clr-1^48^, Clr-2^48^, as well as repressors ACEI^49^ and CreA^50^. Out of these transcription factors, Xyr1, ACEI, ACEII and CreA (termed Cre1 in *H. jecorina*) are present and have been investigated in *H. jecorina*. Interestingly, our previous prediction of phosphorylation sites in these proteins suggested the presence of MAPK phosphorylation sites in all of these transcription factors^5^, leading to the proposal that MAPKs are involved in the regulation of cellulase-coding genes, but which MAPK is involved in regulating the transcription of cellulase-coding genes is unknown. Our investigations on the role of Tmk2 in cellulase production lead to the suggestion that Tmk2 is involved in repressing cellulase production but not transcription (Fig. 3–4). This is in contrast to our previous research on Tmk3^5^ in two aspects. For one, Tmk3 is involved in promoting cellulase production while Tmk2 is involved in repressing cellulase production. For two, Tmk3 regulates cellulase production on the transcription level but not Tmk2. From these results, we can exclude the possibility that Tmk2 phosphorylates these transcription factors. The mechanism by which Tmk2 regulates cellulase production is unknown and requires further investigation, but we can raise a preliminary hypothesis that Tmk2 is involved in post-transcriptional modulation and/or protein synthesis and secretion. More specifically, it is reasonable and promising to propose that the weakened cell wall benefits cellulase secretion in *H. jecorina Δtmk2*, a phenomenon also observed in *H. jecorina Δtmk3* during growth on solid-state media^5^.

There have been many previous reports on factors involved in cellulase formation. Notably most of these identified factors are involved in promoting cellulase formation in filamentous fungi^5^,^51^, rather than suppressing cellulase formation. This is not a surprise because: 1) the cellulase production is a complicated process and therefore requires the involvement of many factors; 2) promoting, rather than repressing cellulase formation is beneficial to filamentous fungi except for in certain circumstances. One of these circumstances is when ‘easier' substances such as glucose are abundant. Under these circumstances, a process termed carbon catabolite repression is triggered, resulting in the inhibition of cellulase formation^52^. The well-known repressor of cellulase-coding gene transcription CreA is involved in this process. Reported in this work is another factor that's shown to repress cellulase production, which is different from previously identified factors on that the regulation doesn't happen on the transcriptional level, therefore providing an additional possibility for engineering *H. jercorina* to improve cellulase production.

Research carried out on Tmk3 in *H. jecorina* revealed one interesting phenomenon: the *Δtmk3* strain grew very poorly on minimal media but only slightly worse than the parent strain on complete media (PDA)^5^. A preliminary hypothesis was made that Tmk3 could be involved in the biosynthesis of certain critical compounds that is not available in minimal media but available in PDA^5^. Unlike *H. jecorina Δtmk3,* although *H. jecorina Δtmk2* doesn't grow as well as the parent strain on both minimal media plates and PDA plates, the growth on minimal media plates was not substantially worse than on PDA plates (Fig. 1c–d), suggesting the involvement of Tmk2 in the same biosynthetic pathway as Tmk3 is unlikely.

Another aspect of fungal physiology in which Tmk2 and Tmk3 show different participation is the resistance to high osmolarity. *H. jecorina Δtmk3* was apparently more sensitive to elevated levels of NaCl in media, suggesting involvement of Tmk3 in resistance of high osmolarity. Results obtained in this work show a different response of *H. jecorina Δtmk2* to higher osmolarity. If *H. jecorina Δtmk2* is more sensitive to high osmolarity, we should be able to see a progressively decreased diameter ratio between *H. jecorina Δtmk2* and parent strains following increased NaCl concentration in media. As is shown in Supplementary Information 3 online and Fig. 5a, what we actually observed is that the diameter ratio between *H. jecorina Δtmk2* and parent strains stayed approximately constant between 0.2 to 1 M NaCl supplemented in the media, therefore suggesting *H. jecorina Δtmk2* is not more sensitive to higher osmolarity in media and Tmk2 is not involved in high osmolarity resistance. The higher diameter ratio between *H. jecorina Δtmk2* and parent strains when media is supplemented with 0.2 M NaCl than 0 M NaCl is an interesting finding. The intracellular osmolarity is similar to that of 0.2 M NaCl. When the media is not supplemented with additional osmotic stabilizers, the cells require the support of intact cell walls to prevent from too much water intake^53^. It can be proposed from these observations that the compromised cell wall in *H. jecorina Δtmk2* couldn't provide the support cells needed to fully survive a low osmolarity environment, and therefore grew more poorly on plates containing no NaCl in comparison to growth on plates containing at least 0.2 M NaCl. This finding is in consistence with, and also provides additional evidence to, our suggestion that Tmk2 is involved in cell wall integrity maintenance.

Besides high osmolarity resistance and biosynthesis in which Tmk3 but not Tmk2 participates in, identified in this work is also one aspect of fungal physiology in which Tmk2 but not Tmk3 is involved. The decreased sporulation level in *H. jecorina Δtmk2* in comparison with the parent strain suggests Tmk2 is involved in sporulation (Fig. 1f). This phenomenon was not observed in *H. jecorina Δtmk3*. Although these results cannot lead to the proposal of specified mechanism by which Tmk2 participates in the sporulation pathway, they are a clear indication that Tmk2 and Tmk3 function differently in this important physiological process.

**Figure 6:**
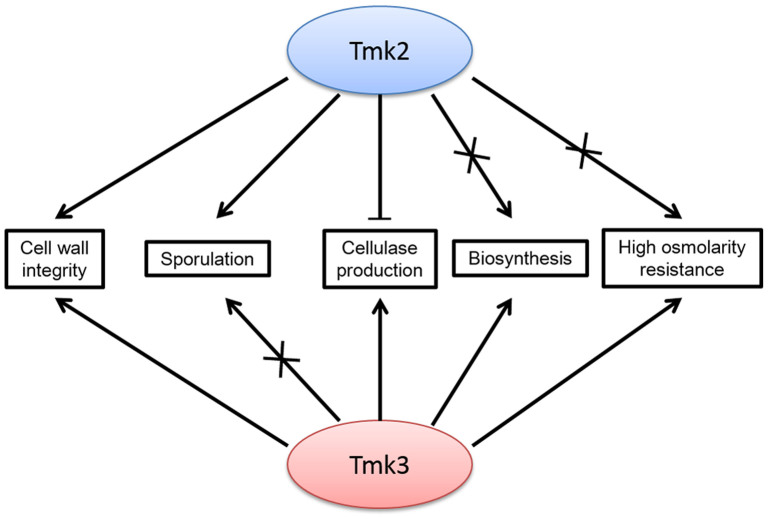
The overlapping and distinct roles of Tmk2 and Tmk3 in *H. jecorina*.

By summarizing the role of Tmk2 identified in this work and comparison with Tmk3, we can clearly see overlapping and distinct functions of Tmk2 and Tmk3 in *H. jecorina* (Fig. 6). They share similar roles in cell wall integrity maintenance, but have distinct functions in high osmolarity resistance, biosynthesis and sporulation. Our most interesting finding is that the deletion of *tmk2* and *tmk3* have drastically different consequences on cellulase production: downregulation for deletion of *tmk3* and upregulation for deletion of *tmk2*. These observations also provide the answers to the questions raised in the beginning of this manuscript: Tmk2 also functions in cell wall integrity maintenance just like the other Slt2-like MAPKs; Tmk2 is far from simply a redundancy to Tmk3 in *H. jecorina*; Tmk2 also participates in cellulase production regulation, but in a different manner. A full scene of the MAPK functions in *H. jecorina* is still not revealed before further work could be carried out on the role of Tmk1, more detailed mechanisms of function for MAPKs, and the relationship between the three MAPKs, which hopefully will help us to more clearly understand fungal physiology in *H. jecorina*, in particular cellulase production and regulation mechanisms.

## Methods

### Strain and chemicals

*H. jecorina Δku70* strain that is a derivative of the *H. jecorina* QM9414 uridine auxotrophic defective TU-6 strain (ATCC MYA-256) was used as the high transformation efficiency parent strain^54^. Wheat bran was a generous gift by Longlive Bio-Technology Co., Ltd., Yucheng, Shandong, China. Uridine was purchased from Sangon Biotech Co., Ltd. (Shanghai, China). Calcofluor white (CFW), *p*NPG, *p*NPC and CMC were purchased from Sigma-Aldrich Corporation (St. Louis, MO, US). Congo red was purchased from Tianjin Damao Chemical Reagent Factory (Tianjin, China). All other chemicals were purchased from Sinopharm Chemical Reagent Co., Ltd. (Shanghai, China).

### Sequence comparison

Sequence alignment between *S. cerevisiae* Slt2, *H. jecorina* Tmk2 and *H. jecorina* Tmk3 was carried out using the Clustal X2 software^55^.

### Construction of *H. jecorina Δtmk2*

To construct *H. jecorina Δtmk2*, a knockout cassette was first constructed which contain the 1.5 kb upstream region of *tmk2*, *pyr4* gene from *H. jecorina*, and 1.5 kb downstream region of *tmk2*. Transformation of the knockout cassette was carried out according to previously published protocols^54^. The transformants were grown on uridine-lacking minimal media plates for selection.

### Growth of *H. jecorina* parent and *Δtmk2* strains on plates

The spores of *H. jecorina* parent and *Δtmk2* strains were first prepared by growing both strains on PDA plates in an incubator (Model DNP-9162, Shanghai Jing Hong Laboratory Instrument Co., Ltd., Shanghai, China) at 30°C and harvesting spores after 7 days. Spores were counted using a hemacytometer. Minimal media solution containing 0.5% NH_4_SO_4_, 0.06% MgSO_4_, 1.5% KH_2_PO_4_, 0.08% CaCl_2_, 0.00005% FeSO_4_·7H_2_O, 0.00016% MnSO_4_·H_2_O, 0.00014% ZnSO_4_·7H_2_O and 0.00002% CoCl_2_ was prepared. Minimal media plates containing 20 ml of minimal media solution, 0.4 g agar, 0.2 g carbon source and 20 mg uridine (parent strain only) were prepared. Avicel double layer plates were prepared as previously described^5^. Approximately 10^5^ spores of *H. jecorina* parent and *Δtmk2* strains were inoculated on PDA plates (4 replicates), Avicel double layer plates (3 replicates) and minimal media plates containing glucose (19 replicates for parent strain, 17 replicates for knockout strain), lactose (3 replicates) or glycerol (3 replicates). These plates except for Avicel double layer plates were incubated in an incubator (Model MJX-250, Ningbo Jiangnan Instrument Factory, Ningbo, China) at 30°C for 66 hours before checking for phenotypic features. The Avicel double layer plates were incubated in the same incubator at 30°C for 5 days prior to examination.

### Microscopic observations

PDA agar plates were first prepared, from which a thin slice (~1 mm thick) of the media was cut off and laid carefully on top of a glass slide. A cover slide was then put on top of the media, creating a ‘sandwich'. A drop of water containing approximately 10^5^ spores of *H. jecorina* parent or *Δtmk2* strains was subsequently inoculated on the side of the cover slide. The inoculated sandwich was then transferred to a glass plate in which a piece of cotton rinsed with 30% glycerol was present to maintain moisture. The plate was then incubated at room temperature for 42 hours. The glass slides were then removed from the glass plates for microscopic observation with a bright field microscope (Nikon eclipse E100, 400 fold magnification).

### Sensitivity to CR, CFW, NaCl and H_2_O_2_

Approximately 10^5^ spores of *H. jecorina* parent and *Δtmk2* strains were inoculated on PDA plates containing 25, 50, 75, 100, 150, 200 μg/ml CR, 20, 40, 60, 80 μg/ml CFW, 0.2, 0.4, 0.6, 0.8, 1.0 M NaCl, or 1, 2, 3, 4 mM H_2_O_2_. These plates were grown at 30°C in an incubator (Model MJX-250, Ningbo Jiangnan Instrument Factory, Ningbo, China) for 66 hours prior to examination. The diameters of colonies on each plate were measure for comparison. Three replicates of each experiment were performed.

### Assay for sporulation

Approximately 10^5^ spores of *H. jecorina* parent and *Δtmk2* strains were inoculated on PDA plates and grown at 30°C in an incubator (Model MJX-250, Ningbo Jiangnan Instrument Factory, Ningbo, China) for 6 days until maximum sporulation was achieved. The spores were subsequently washed with washing solution (0.9% NaCl plus 0.05% Tween-80) and counted with a hemacytometer. Three biological replicates and three technical replicates for each biological replicate (a total of 9 replicates) were carried out for each strain.

### Biochemical assays

*H. jecorina* parent and *Δtmk2* strains were grown in liquid media containing 100 ml of minimal media solution, 2 g of wheat bran, 2 g of Avicel and 100 mg uridine (parent strain only). Approximately 10^6^ spores were inoculated in liquid media to initiate growth. The cultures were shaken in a shaker (Model SKY-1112B, Shanghai Sukun Ltd., Shanghai, China) at 30°C and 200 rpm. Aliquots were drawn each day for biochemical assays. Tests for FPA, *p*NPCase, *p*NPGase, CMCase activities, extracellular protein levels and total ATP content were carried out essentially as previously described^5^. Three biological replicates for each activity were carried out.

### Real-time PCR reactions

Real-time PCR (qPCR) reactions were carried out under identical conditions as in previous work on Tmk3^5^. The reactions start with a preincubation step at 95°C for 300 seconds, followed by 40 cycles of three-step amplification (10 seconds at 95°C, 10 seconds at 59°C, 10 seconds at 72°C) and a melting step (10 seconds at 95°C, 60 seconds at 65°C, 1 second at 97°C). Data analysis was carried out using Microsoft Excel. Primers and amplicon sizes are indicated in Supplementary Information 4 online.

For evaluation of transcriptional abundance of chitin synthase- and β-1,3-glucan synthase-coding genes, approximately 10^6^ spores of *H. jecorina* parent and *Δtmk2* strains were inoculated in liquid media containing 100 ml minimal media solution, 2 g glucose and 100 mg uridine (parent strain only). The cultures were shaken in a shaker (Model SKY-1112B, Shanghai Sukun Ltd., Shanghai, China) at 30°C and 200 rpm for 3 days. The cultures were subsequently harvested for total RNA extraction. The cDNA was synthesized using PrimeScript^TM^ RT reagent kit with gDNA eraser (Perfect Real Time) from Takara Bio Inc. (Shiga, Japan). qPCR reactions were carried out on a LightCycler 96 Real-Time PCR system (Roche Applied Science, Mannheim, Germany) using FastStart Essential DNA Green Master (Roche Applied Science, Mannheim, Germany) as the dye. Three individual biological replicates and three individual technical replicates for each biological sample (a total of 9 replicates for each reaction) were carried out.

For evaluation of transcriptional levels of cellulase-coding genes and transcription factor-coding genes, *H. jecorina* parent and *Δtmk2* strains were grown essentially as described above except for the inclusion of 2 g wheat bran and 2 g Avicel instead of glucose in the media. The synthesis of cDNA and performance of qPCR reactions were carried out similarly as described above. Nine biological replicates and three technical replicates for each biological replicate were carried out for cellulase-coding genes and transcription factor-coding genes (a total of 27 replicates).

### Statistical analysis

For tests of significant difference between two sets of data, two-tailed Student's *t*-tests were carried out. P<0.05 was considered significant different. A minimum of three replicates were present in each set of data.

## Author Contributions

M.W., Y.D., Q.Z., K.L. and B.J. constructed the knockout strain and carried out phenotypic analyses. Y.D., Q.Z. and F.W. carried out qPCR analyses. M.W. and X.F. conceived of the study, interpreted data, prepared figures and wrote the manuscript. All authors reviewed the manuscript. X.F. oversaw and coordinated the project.

## Supplementary Material

Supplementary Information Supplementary information
