# Supplementary material for: Identification of the role of a MAP kinase Tmk2 in Hypocrea jecorina (Trichoderma reesei)
Source: Sci Rep. 2014 Oct 23;4:6732. doi: 10.1038/srep06732 (PMC4206845; doi:10.1038/srep06732)
Supplement: Supplementary Information [file srep06732-s1.pdf]

Identification of the role of a MAP kinase Tmk2 in *Hypocrea jecorina* (*Trichoderma reesei*)

Mingyu Wang [1,2], Yanmei Dong [1], Qiushuang Zhao [1], Fangzhong Wang [1], Kuimei Liu[1], Baojie Jiang [1], Xu Fang [1,2,*]

1. State Key Laboratory of Microbial Technology, School of Life Sciences, Shandong University, Jinan 250100, China

2. National Glycoengineering Research Center, Shandong University, Jinan 250100, China

[*] To whom correspondence is addressed. E-mail: fangxu@sdu.edu.cn

Tel: +86-531-88364004, Fax: +86-531-88364363

Supplementary Information 1-4

Supplementary Information 1. Sequence alignment between Tmk2 from *H. jecorina* and Slt2 from *S. cerevisiae*

```
Tmk2            MADLQGRKVFKVFNQDFVVDERYTVTKELGQGAYGIVCAAVNNQTNEG--VAIKKVTNVF
Slt2            MADKIERHTFKVFNQDFSVDKRFQLIKEIGHGAYGIVCSARFAEAAEDTTVAIKKVTNVF
                ***   *:.******** **:*: : **:*:*******:*   :: *.  **********

Tmk2            SKKILAKRALREIKLLQHFRGHRNITCLYDMDIP-RPD-NFNETYLYEELMECDLAAIIR
Slt2            SKTLLCKRSLRELKLLRHFRGHKNITCLYDMDIVFYPDGSINGLYLYEELMECDMHQIIK
                **.:*.**:***:***:*****:**********   ** .:*  **********:  **:

Tmk2            SGQPLTDAHFQSFIYQILCGLKYIHSANVLHRDLKPGNLLVNADCELKICDFGLARGFSV
Slt2            SGQPLTDAHYQSFTYQILCGLKYIHSADVLHRDLKPGNLLVNADCQLKICDFGLARGYSE
                *********:*** *************:*****************:***********:* 

Tmk2            DPEENAGYMTEYVATRWYRAPEIMLSFQSYTKAIDVWSVGCILAELLGGRPFFKGRDYVD
Slt2            NPVENSQFLTEYVATRWYRAPEIMLSYQGYTKAIDVWSAGCILAEFLGGKPIFKGKDYVN
                :* **: ::*****************:*.*********.******:***:*:***:***:

Tmk2            QLNQILHILGTPNEETLRRIGSPRAQEYVRNLPFMPKKPFPALFPDANPDALDLLDKMLA
Slt2            QLNQILQVLGTPPDETLRRIGSKNVQDYIHQLGFIPKVPFVNLYPNANSQALDLLEQMLA
                ******::**** :******** ..*:*:::* *:** **  *:*:**.:*****::***

Tmk2            FDPSQRISVEQALEHPYLHIWHDASDEPDCPTTFNFDFEVVEDVGEMRKMILDEVLRFRQ
Slt2            FDPQKRITVDEALEHPYLSIWHDPADEPVCSEKFEFSFESVNDMEDLKQMVIQEVQDFRL
                ***.:**:*::******* ****.:*** *. .*:*.** *:*: ::::*:::**  ** 

Tmk2            LVRT------------APASGNQGAGQQIQVPLPSAGGQWTAED----------------
Slt2            FVRQPLLEEQRQLQLQQQQQQQQQQQQQQQPSDVDNGNAAASEENYPKQMATSNSVAPQ
                :**                . :*   ** * *    .*: :*.:

Tmk2            -------------------PRPQEYMGQP---------------NG----LEQELQAGMD
Slt2            QESFGIHSQNLPRHDADFPPRPQESMMEMRPATGNTADIPPQNDNGTLLDLEKELEFGLD
                                   ***** * :                **    **:**: *:*

Tmk2            IRR-
Slt2            RKYF
                 :
```

Supplementary Information 2. Sequence alignment between Tmk2 and Tmk3 from *H. jecorina*.

```
Tmk2            MADLQGRKVFKVFNQDFVVDERYTVTKELGQGAYGIVCAAVNNQTNEGVAIKKVTNVFSK
Tmk3            MAEFVR---AQIFGTTFEITSRYSDLQPVGMGAFGLVCSARDQLTNQNVAVKKIMKPFST
                **::      ::*.  * : .**:  : :* **:*:**:* :: **:.**:**: : **.

Tmk2            KILAKRALREIKLLQHFRGHRNITCLYDMDIPRPDNFNETYLYEELMECDLAAIIRSGQP
Tmk3            PVLAKRTYRELKLLKHLR-HENVISLSDIFISP---LEDIYFVTELLGTDLHRLLTS-RP
                 :****: **:***:*:* *.*: .* *: *.    ::: *:  **:  **  :: * :*

Tmk2            LTDAHFQSFIYQILCGLKYIHSANVLHRDLKPGNLLVNADCELKICDFGLARGFSVDPEE
Tmk3            LEKQFIQYFLYQIMRGLKYVHSAGVVHRDLKPSNILVNENCDLKICDFGLAR--IQDPQ-
                * . .:* *:***: ****:***.*:******.*:*** :*:**********    **:

Tmk2            NAGYMTEYVATRWYRAPEIMLSFQSYTKAIDVWSVGCILAELLGGRPFFKGRDYVDQLNQ
Tmk3            ----MTGYVSTRYYRAPEIMLTWQKYDVEVDIWSAGCIFAEMLEGKPLFPGKDHVNQFSI
                    ** **:**:********::*.*   :*:**.***:**:* *:*:* *:*:*:*:.

Tmk2            ILHILGTPNEETLRRIGSPRAQEYVRNLPFMPKKPFPALFPDANPDALDLLDKMLAFDPS
Tmk3            ITELLGTPPDDVINTIASENTLRFVKSLPKRERQPLRNKFKNADDSAVDLLERMLVFDPK
                * .:**** ::.:. *.* .: .:*:.**   ::*:   * :*: .*:***::**.***.

Tmk2            QRISVEQALEHPYLHIWHDASDEPDCPTTFNFDFEVVED-VGEMRKMILDEVLRFRQLVR
Tmk3            KRITATEALAHEYLAPYHDPTDEPVAEEKFDWSFNDADLPVDTWKIMMYSEILDYHNIEG
                :**:. :** * **  :**.:*** .  .*::.*: .:  *.  : *: .*:* ::::

Tmk2            TAPASGNQGAGQQIQVPLPSAGGQWTAEDPRPQEYMGQPNGLEQELQAGMDIRR
Tmk3            GVPNMDEQFPPQ------------------------------------------
                 .*  .:* . *
```

Supplementary Information 3. Colony diameters of *H. jecorina* parent and *Δtmk2* strains grown on plates. All values are expressed in mean ± s.d. (n=4 for PDA plates, n=19 for *H. jecorina* parent strain grown on glucose plates, n=17 for *H. jecorina Δtmk2* grown on glucose plates, n=3 for all other plates).

Plates	Parent (mm)	*Δtmk2* (mm)	Plates	Parent (mm)	*Δtmk2* (mm)
PDA	78.0 ± 0.8	28.5 ± 2.1	PDA+1 mM H_2O_2	63.0 ± 5.0	28.3 ± 7.5
PDA+0.2 M NaCl	59.7 ± 5.8	30.3 ± 0.6	PDA+2 mM H_2O_2	46.3 ± 10.2	22.3 ± 1.2
PDA+0.4 M NaCl	43.3 ± 2.9	27.0 ± 1.0	PDA+3 mM H_2O_2	0.0 ± 0.0	0.0 ± 0.0
PDA+0.6 M NaCl	34.3 ± 2.1	24.3 ± 0.6	PDA+4 mM H_2O_2	0.0 ± 0.0	0.0 ± 0.0
PDA+0.8 M NaCl	23.3 ± 0.6	18.3 ± 1.2	Lactose	33.0 ± 1.0	23.7 ± 0.6
PDA+1.0 M NaCl	14.0 ± 0.0	11.3 ± 0.6	Glucose	48.4 ± 2.5	21.5 ± 0.9
Glycerol	23.0 ± 1.7	16.7 ± 0.6	Avicel	59.3 ± 1.5	37.3 ± 1.1

Supplementary Information 4. Primers used in real-time PCR reactions and amplicon sizes.

Primer	Sequence (5' to 3')	Amplicon size (bp)
actin-F	TTAAGAAAGCCGCCACCCCC	134
actin-R	GTTGGTCGACAGGGAGAGGATG	
cbh1-F	CTGCGACTGGAACCCATACC	132
cbh1-R	AAGTGACGCCATTCTGGACAT	
cbh2-F	CGTTGCTGGATTCGTTTGTC	178
cbh2-R	GATGGGTTTGCGTTTGTGAG	
egl1-F	CCCGAGGTCCATCCCAAG	100
egl1-R	TCCAGCGGTAGTTCCAGTCAA	
egl2-F	CAACGACGACGGGATGACTA	246
egl2-R	GATGCCAACTGCGACCAAA	
bgl1-F	TGGGCGGGTCTTCCTTC	104
bgl1-R	GGGCTCTTCGCAATGGTGTA	
xyr1-F	ACAGTGGAGCGGTAACAGACA	209
xyr1-R	CACGAATCCTTCCGACGAG	
cre1-F	CTCCTTCTTCTCCCACCTT	140
cre1-R	CTGCAGGGACAGGTTTCT	
ace1-F	AGACCCTGATCTTCATGGCAC	110
ace1-R	GTCGGTGAGATATTCGACTGT	
ace2-F	TCAACATCCTCCACCACCAG	117
ace2-R	CTTGAGACGTGATGCATGTC	
Trire2_112271-F	TCTTCATCGCAGTCAAGGCCATC	204
Trire2_112271-R	TAGGCGTCAGGAGCATGTACTGG	
Trire2_58188-F	GCGAACCTTCCCCAAGAGAAAGC	251
Trire2_58188-R	CGACATAGAAGGTGAAGGCGA	
Trire2_55341-F	CCATGGACCGTTGAGCCAATACTT	203
Trire2_55341-R	AGATGAACTCGGGGACGGAATCG	
Trire2_51492-F	ATGGCTACAAGTCCTTCCGTACC	118
Trire2_51492-R	GAAGCAGTTCCCACGAAACCAGAC	
Trire2_124228-F	TAGTGACGCTACCACTTCCGAGC	266
Trire2_124228-R	CACCGAGTTGATGGCCGTGAT	
Trire2_67600-F	CAACGACGTGATGACCCAGCA	195
Trire2_67600-R	TCGTTGGAGATGCTACCGAGG	
Trire2_122172-F	ATGGTTGGCCTTCCGAGCGA	172
Trire2_122172-R	GGAGAGCTTCGGTGGCACTATTG	

Trire2_71563-F	TGCGTTGTCTTGGGCTCTCTG	194
Trire2_71563-R	AGGTTCAGAGACAGAGTCACGCC	
Trire2_122993-F	AATGTCGTCGGCTGCCTACTC	206
Trire2_122993-R	TCATCGCTCCTCTCATCCCGT	
fks-F	CAACCAGAAGCCTACCGACTCTC	225
fks-R	CCCTCGAGCAAGAAGAGCACT	
